# A retrospective review to identify criteria for incorporating the Singapore flap and gracilis muscle flap into obstetric fistula repair

**DOI:** 10.1002/ijgo.13038

**Published:** 2020-01-13

**Authors:** Rachel Pope, Pierce C. Hollier, Rodger H. Brown, Chisomo Chalamanda, Larry H. Hollier, Jeffrey Wilkinson

**Affiliations:** ^1^ Division of Global Women's Health Department of Obstetrics and Gynecology Baylor College of Medicine Houston TX USA; ^2^ School of Medicine Duke University Durham NC USA; ^3^ Division of Plastic Surgery Department of General Surgery Houston Methodist Houston TX USA; ^4^ Freedom from Fistula Fistula Care Centre Lilongwe Malawi; ^5^ Division of Plastic Surgery Baylor College of Medicine Houston TX USA

**Keywords:** Fistula repair, Global women's health, Gracilis muscle, Obstetric fistula, Singapore flap, Surgical collaboration, Vesicovaginal fistula

## Abstract

**Objective:**

To identify criteria to guide surgeons regarding indications for use of the Singapore and gracilis muscle flaps in obstetric fistula repair.

**Methods:**

This is a retrospective case series. Obstetric fistula surgeons in Lilongwe, Malawi, have been incorporating plastic surgery techniques with the Singapore and gracilis muscle flaps since collaborating with plastic surgeons in 2016. We describe the surgical outcomes of procedures utilizing each flap individually and those using both.

**Results:**

Between February 2016 and June 2019, 69 patients received a flap at the time of obstetric fistula repair at the Fistula Care Center in Lilongwe, Malawi. A total of 32 (46.4%) received a Singapore flap, 20 (29.0%) received a gracilis flap, and 17 (24.6%) received both types of flap.

**Conclusion:**

Based on our outcomes, we note the possible advantage of incorporating the gracilis flap even when it is thought that the Singapore flap is sufficient. However, more data are needed.

## Abstract 2

Based on our retrospective review, we suggest criteria for using the Singapore and gracilis muscle flaps in complex obstetric fistula repair.

## 1 INTRODUCTION

Since 2016, surgeons at the Fistula Care Centre (FCC) in Lilongwe, Malawi, have been incorporating Singapore fasciocutaneous flaps and gracilis muscle flaps for complex obstetric fistula repairs. This development arose from a collaboration with plastic surgeons out of the need for additional techniques for complex repairs. Since then, we have performed approaching 70 fistula repairs incorporating one or both flaps. We have been encouraged by the outcomes as these are the most complex fistulae to repair—quoted to have as low as a 52% success rate.^1^

**Figure 1:**
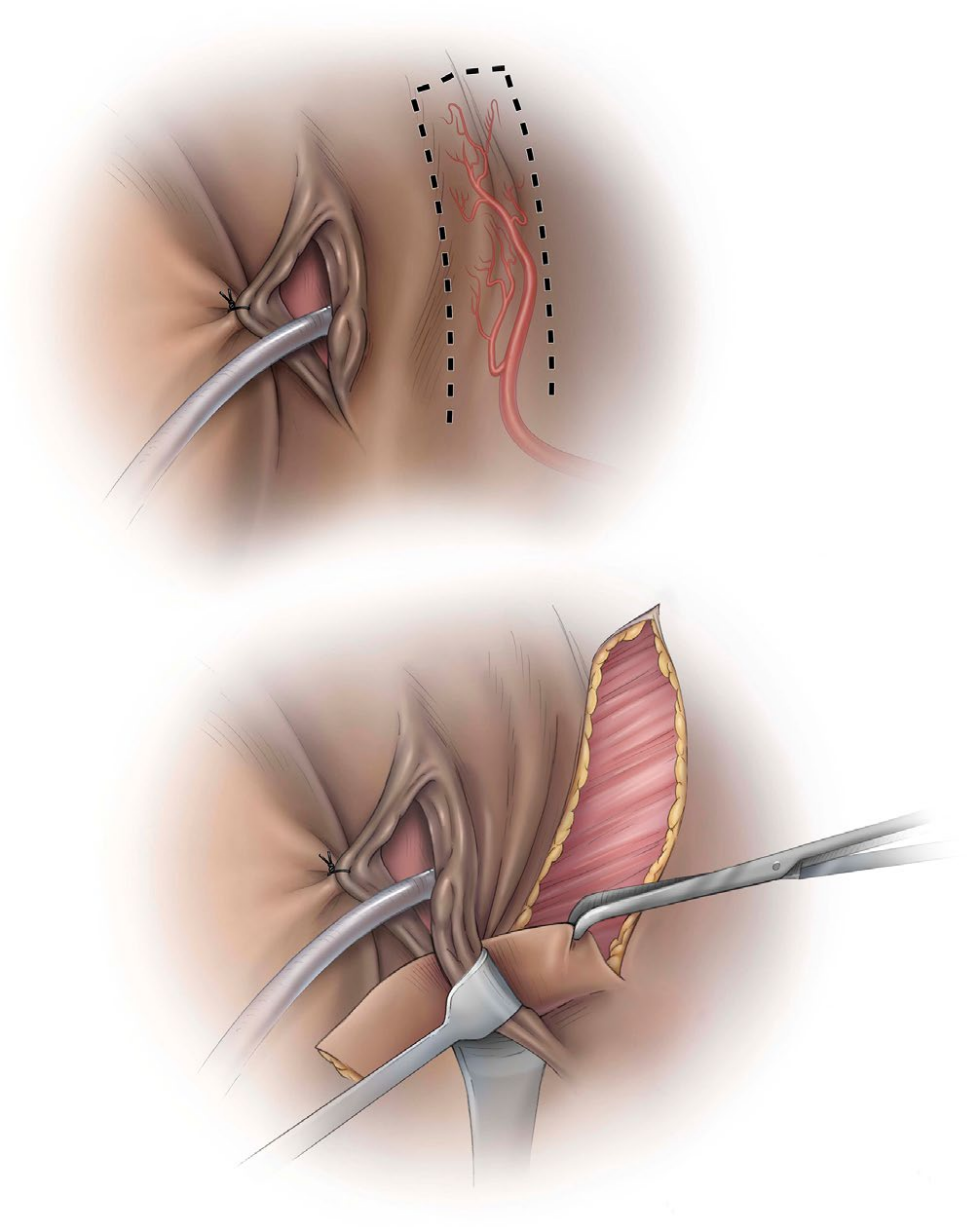
Harvesting the Singapore flap to include the posterior labial arterial supply. © Baylor College of Medicine. Image created by Katherine Relyea, MS, CMI and printed with permission from Baylor College of Medicine.

**Figure 2:**
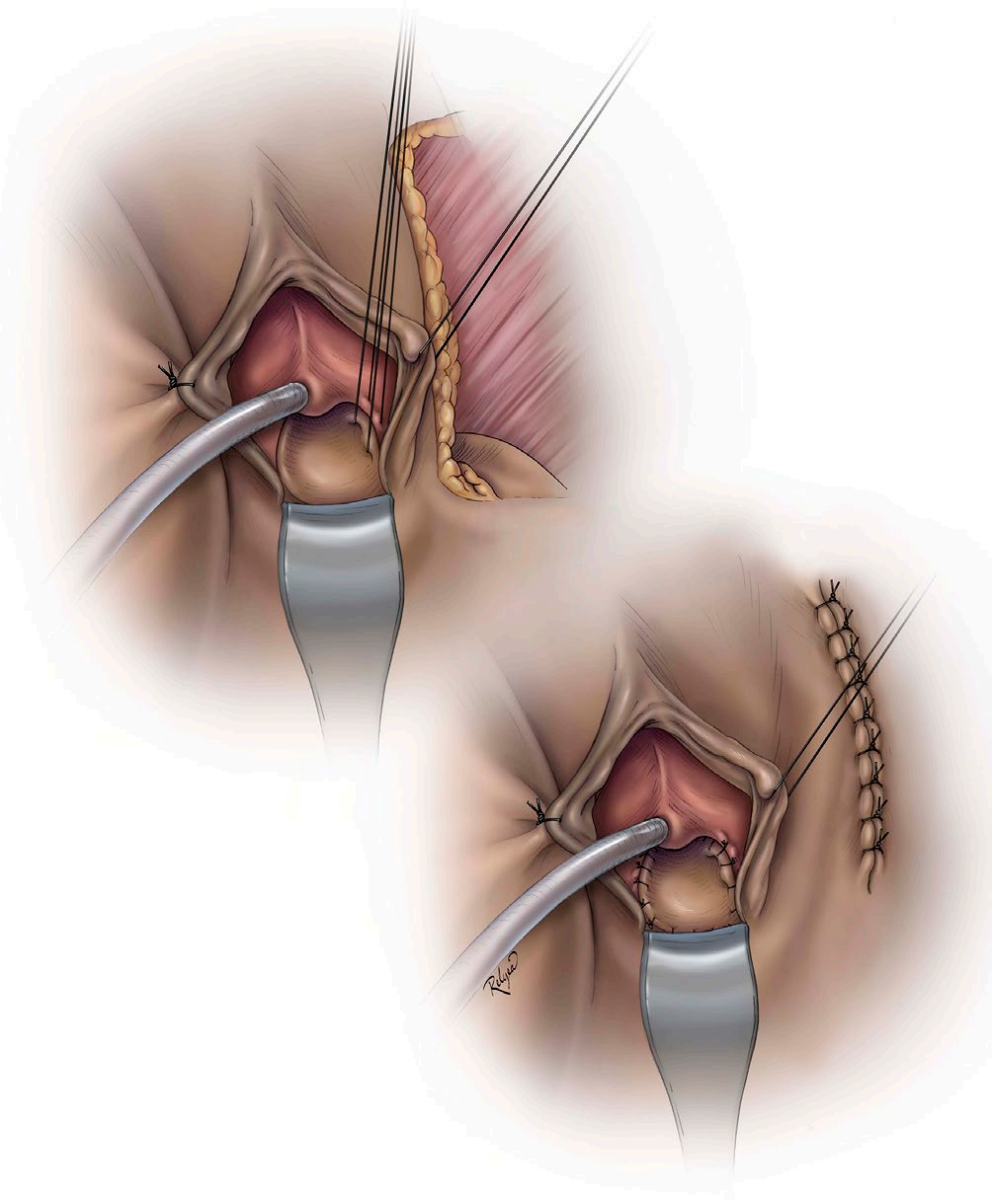
Using the Singapore flap in the posterior vagina (can also be used anteriorly). © Baylor College of Medicine. Image created by Katherine Relyea, MS, CMI and printed with permission from Baylor College of Medicine.

**Figure 3:**
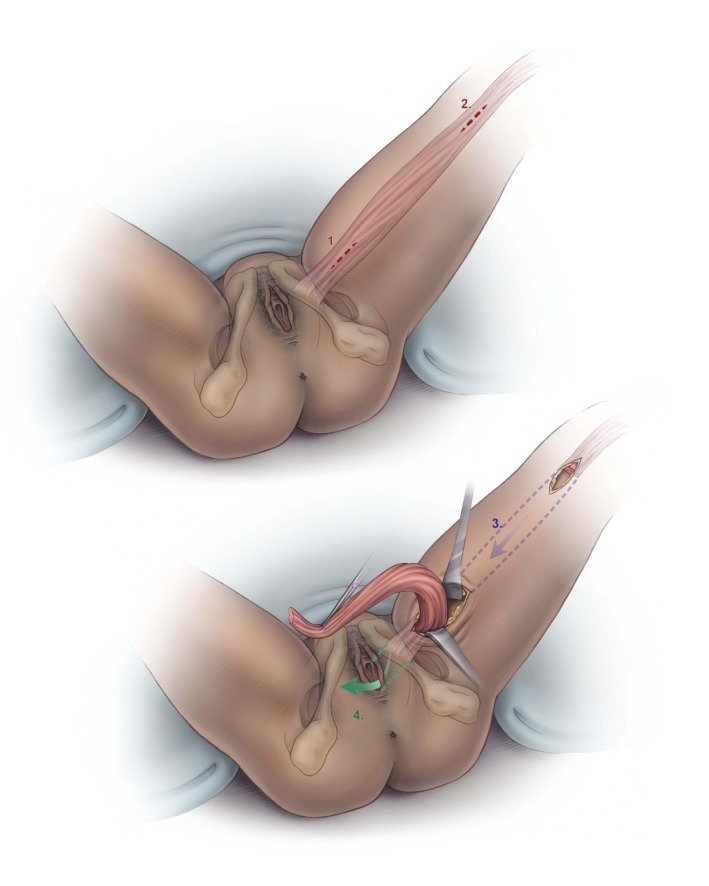
Harvesting the gracilis muscle flap. © Baylor College of Medicine. Image created by Katherine Relyea, MS, CMI and printed with permission from Baylor College of Medicine.

**Figure 4:**
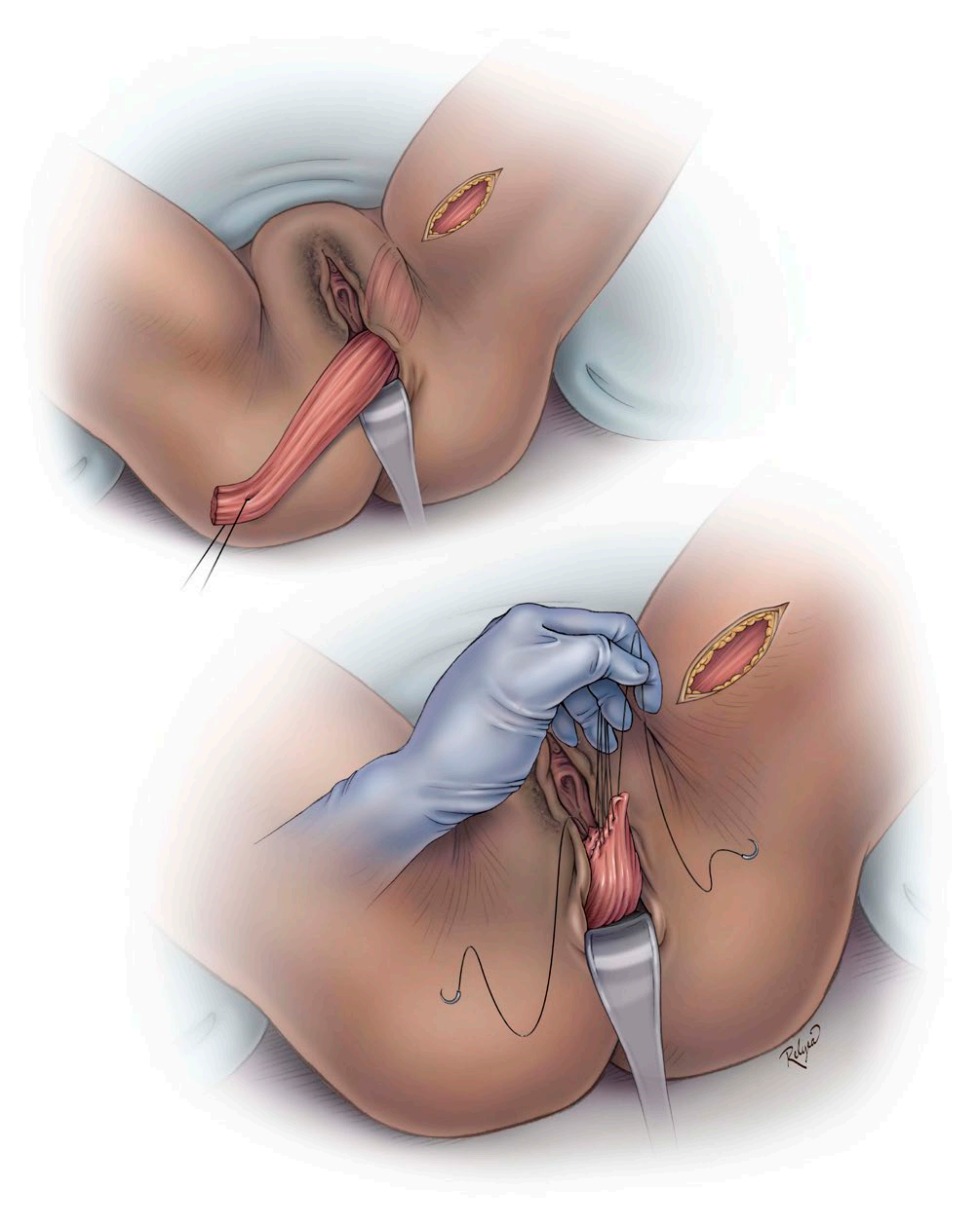
Tunneling the muscle into the vagina. © Baylor College of Medicine. Image created by Katherine Relyea, MS, CMI and printed with permission from Baylor College of Medicine.

We have since held two workshops at the FCC along with a plastic surgeon (RB), during which we taught other fistula surgeons the flap techniques (Figs 1, 2, 3, 4). For surgical techniques on flap creation, please see our papers on the Singapore flap for vaginal reconstruction and the gracilis muscle flap for complex repairs.^2^, ^3^ From the second larger workshop we found that indications for use of the flaps are subjective. Therefore, the aim of the present study was to review our outcomes to date and attempt to determine criteria to guide others in the use of these flaps.

## 2 MATERIALS AND METHODS

This is a retrospective review of all obstetric fistula repairs that used a Singapore flap, gracilis muscle flap, or both. Cases using a flap for vaginal reconstruction without the presence of a fistula were not included in this review. This is part of a larger study on the outcomes of obstetric fistula repair at our center, which has been approved by the Malawian National Health Sciences Research Committee and the Baylor College of Medicine Institutional Review Board. All cases in which a flap was incorporated into the obstetric fistula repair were included in the cohort. Trained research assistants double‐entered and compared the data using RedCap (Research Electronic Data Capture, Vanderbilt University, Nashville, TN, USA) and a physician cleaned all data.

The variables we examined included patient age, fistula duration, Goh classification of the fistula injury,^4^ number of previous repairs, type of physical characteristics of the injury, outcomes of the repair, and operative or postoperative complications. At our center, we conduct a dye test at the time of catheter removal. If the dye test is positive but there is no obvious breakdown of the fistula repair and the Foley catheter is still draining urine, we keep the catheter in place for one additional week. If the subsequent dye test remains positive, or if at the first dye test the repair has grossly broken down and the Foley catheter is not draining urine, we consider this a failed repair. For patients with successful closure of the fistula, a 1‐hour pad weight test is done to quantify the amount of urethral leakage; this is because many women with a complex fistula experience some degree of urethral leakage postoperatively despite a healed fistula.

## 3 RESULTS

A total of 69 patients received a flap or combination of flaps during fistula repair at the Fistula Care Center in Lilongwe, Malawi between February 2016 and June 2019. In all, 32 (46.4%) women received a Singapore flap, 20 (29.0%) received a gracilis flap, and 17 (24.6%) received both types of flap. Table 1 outlines the variables of interest in forming criteria to guide employment of each flap.

**Table 1:** Characteristics of fistula repair case (n=69) based on flap type.^a^

Characteristics	Singapore flap (n=32)	Gracilis flap (n=20)	Both Singapore and gracilis flaps (n=17)	*P* value
Age, y	27.2 ± 9.0	42.4 ± 17.7	35.2 ± 14.9	0.009
Duration of fistula, y	3.0 ± 3.6 (n=30)	10.4 ± 10.2 (n=14)	8.2 ± 6.4 (n=9)	0.002
Mode of delivery	(n=32)	(n=19)	(n=16)	0.546
Spontaneous vaginal	18 (56.3)	8 (42.1)	7 (43.7)	
Cesarean	14 (43.7)	11 (57.9)	9 (56.3)	
Type of initial injury (Goh classification)				
Proximity to the urethra				
Type 1	2 (6.3)	2 (10.0)	0	0.440
Type 2	4 (12.5)	1 (5.0)	0	
Type 3	17 (53.1)	9 (45.0)	8 (47.1)	
Type 4	9 (28.1)	8 (40.0)	9 (52.9)	
Size				
A	5 (15.6)	4 (20.0)	1 (5.9)	0.663
B	7 (21.9)	6 (30.0)	5 (29.4)	
C	20 (62.5)	10 (50.0)	11 (64.7)	
Scar tissue				
i	2 (6.3)	1 (5.0)	0	0.298
ii	5 (15.6)	1 (5.0)	1 (5.9)	
iii	25 (78.1)	18 (90.0)	16 (94.1)	
History of a previous surgical attempt	10 (31.3)	10 (50)	6 (35.3)	0.387
Urethral length, cm	1.88 ± 0.76 (n=30)	1.55 ± 0.72	1.41 ± 0.82 (n=16)	0.1023
Bladder length, cm	7.20 ± 1.59 (n=30)	6.35 ± 1.97	6.13 ± 1.77 (n=16)	0.0933
Vaginal length, cm	6.17 ± 1.90 (n=30)	7.18 ± 0.89	5.56 ± 2.34 (n=16)	0.0268
Degree of vaginal scarring				
None	4 (14.8)	3 (17.7)	1 (6.3)	0.119
Minimal	2 (7.4)	3 (17.7)	2 (12.5)	
Moderate	14 (51.9)	11 (64.7)	7 (43.8)	
Severe	11 (40.7)	2 (11.8)	4 (25.0)	
Obliterated	0 (0.0) (n=27; 1=N/A)	1 (5.9) (n=17)	3 (18.8) (n=16)	
Dye test negative	22 (68.8)	18 (90.0)	12 (70.6)	0.167
Cough test negative	9 (32.1) (n=28)	6 (30.0)	3 (23.1) (n=13)	0.838
Pad weight, g	26.5 ± 21.2 (n=25)	21.5 ± 29.3 (n=19)	21.1 ± 18.6 (n=12)	0.7255
Number of days of catheterization	20.0 ± 6.0	19.3 ± 5.6	22.4 ± 4.5	0.2093
Postoperative complication	13 (40.6)	7 (35.0)	8 (47.1)	0.758

a Values are given as number (percentage) and mean ± SD unless otherwise indicated.

### 3.1 Singapore flap

The mean age of the women who received a Singapore flap was 27.2 ± 9.0 years; these women had lived with a fistula for an average of approximately 3 years. About half had a spontaneous vaginal delivery. Fistula injuries incurred based on the Goh classification were primarily type 3 and 4 (indicating maximum involvement of the urethra), mostly larger than 3 cm in diameter, and were type iii indicating significant scarring, previous repair, or were circumferential in nature. Ten (31%) women had undergone a previous repair. Average urethral length was 1.88 ± 0.76 cm, bladder length 7.20 ± 1.59 cm, and vaginal length 6.17 ± 1.90 cm. Scarring was considered moderate to severe in most cases. Dye tests were negative in 22 (68.8%) women. Nine (32.1%) women did not leak urine from the urethra on the cough test, indicating complete “dryness.” This was measured objectively using a 1‐hour pad weight test. Mean pad weight was 26.5 ± 21.2 g. Thirteen (40.6%) women experienced a postoperative complication, which was primarily minor wound breakdown from the donor site.

### 3.2 Gracilis flap

A gracilis flap was used during fistula repair in 20 women. Average age was 42.4 ± 17.7 years. Many had lived with a fistula for several years: 10.4 ± 10.2 years on average. Eleven (57.8%) women had undergone cesarean delivery at the delivery that led to an obstetric fistula. Most women (85%) had a Goh type 3 or 4 injury indicating maximum involvement of the urethra. Half were large fistulae greater than 3 cm, and 90% were type iii due to scarring, previous repair, or were circumferential in nature. Ten (50.0%) women had undergone a previous repair attempt. Mean urethral length was 1.55 ± 0.72 cm, bladder length 6.35 ± 1.97 cm, and vaginal length 7.18 ± 0.89 cm. Most had a moderate degree of scarring (n=11; 64.7%), but few had severe scarring or an obliterated vagina. Eighteen (90.0%) women healed successfully and six (30.0%) were completely dry with no urethral leakage. Average pad weight was 21.5 ± 29.3 g. Seven (35.0%) women had a postoperative complication, mostly urinary tract infections (n=6), and one blocked catheter (n=1).

### 3.3 Both flaps

Seventeen (24.6%) women had both types of flaps at the time of fistula repair. Average age of the women was 35.2 ± 14.9 years, and they had spent an average of 8.2 ± 6.4 years with a fistula. Nine (56.3%) women had undergone cesarean delivery at the time of the fistula injury. Most were Goh type 3 and 4, greater than 3 cm in diameter, and 94.1% (n=16) were classified as type iii, due to scarring, previous repair, or were circumferential in nature. Six (35.3%) women had had a previous repair attempt. Average urethral length was 1.41 ± 0.82 cm, bladder length 6.13 ± 1.77 cm, and vaginal length 5.56 ± 2.34 cm. Most women had moderate to severe scarring. Only 10 (58.8%) of these cases had a negative dye test initially and 3 (23.1%) were completely dry with no urethral leakage. However, two patients returned for follow‐up with a negative dye test, increasing the success rate to 70.6% (n=12). Average pad weight was 21.1 ± 18.6 g. Eight (47.1%) patients had a postoperative complication, mostly due to minor wound breakdowns.

## 4 DISCUSSION

The literature reveals that, overall, outcomes for cases including flaps are better than for complex fistula repairs without flaps. Singapore flaps are employed in cases of moderate to severe scarring where there is a compromise of vaginal length, likely due to a large fistula. Gracilis flaps are employed when there has been a previous repair attempt, the fistula injury is severe, but vaginal reconstruction is not required. Both flaps are employed when the injury is severe and vaginal reconstruction is required owing to a short length or severe scarring.

Incorporating a gracilis flap even in instances where the Singapore flap is felt to be sufficient is likely warranted given our lowest success rate for continence when using the Singapore flap alone. Although the Singapore flap carries a vascular supply, it is less robust than that of the gracilis muscle and is technically easier to compromise. Improving blood supply in compromised areas is paramount in any reconstructive procedure. Augmentation of the Singapore flap with the vascularity of the gracilis is beneficial to suture line healing and minimizing contracture. This is most poignantly demonstrated in two patients who had both a Singapore flap and a gracilis flap, who were discharged initially with a failed repair but returned to the center healed.

Our incontinence outcomes with the Singapore flap are lower than with the gracilis flap, suggesting again that perhaps the gracilis flap should be used more often in repairing large fistulae even when it seems that the Singapore flap is adequate. We have found that the Singapore flap greatly improves quality of life for women who need vaginal reconstruction. However, in the case of large complex fistulae, it may not be enough.

All cases involved a short urethra. A suboptimal number of patients had no urethral leakage, indicating that more efforts are needed to develop anti‐incontinence techniques. This has been found in the literature for complex fistulae, namely Goh types 3 and 4 and represents the perpetual continence gap.^5^ Of note, the highest success rate was for women who had the gracilis flap alone (90%, n=18). However, this does not necessarily mean that a gracilis flap and a Singapore flap together are not recommended, as these cases may be considered “the worst of the worst.” The cases in which both flaps were employed were, on average, the most severe types according to the Goh classification. They also had, on average, the shortest urethras and the shortest vaginal lengths. This is consistent with the use of the Singapore flap when vaginal reconstruction is required along with a complex fistula injury.

Browning et al.^6^ describe the Singapore flap as a solution to prevent urethral incontinence by preventing contraction of tissue that would draw the urethra open. Unfortunately, many women in this cohort still experienced urethral incontinence with the Singapore flap and average pad weights were still high for this high‐risk group.

The strengths of the present study are the large number of complex cases and the ability to follow outcomes because we were operating in a national referral center for women with obstetric fistulae. The major limitation is the retrospective nature of these data and the small sample size. However, it is exceedingly difficult to randomize cases with such heterogeneity of injury and these are relatively new techniques for use in obstetric‐related fistula repairs. Future prospective studies, however, could use our suggested criteria in following outcomes.

In summary, we suggest the Singapore flap be used for vaginal reconstruction when large fistula injury has caused vaginal length shortening and/or severe tissue loss. We suggest the gracilis flap alone in cases of complex injury and previously failed repair attempts without vaginal tissue compromise. Finally, both flaps should be used when all of these variables are present, and perhaps more often in order to gain improved closure rates in all cases. We next plan to follow the quality of life and long‐term outcomes including pad weights and urethral leakage to understand the changes over time. Ongoing work to decrease urethral leakage is still necessary to improve the impact of surgical repair on overall continence.

## AUTHOR CONTRIBUTIONS

RP came up with the concept and initiated this study, drafted the manuscript and finalized edits. PC collected data and edited the manuscript. RP, RB, CC, LH, and JW contributed to the manuscript editing and surgical procedures performed in the study.

## CONFLICTS OF INTEREST

The authors have no conflicts of interest.
